# Supplementary material for: Evidence supported by Mendelian randomization: impact on inflammatory factors in knee osteoarthritis
Source: Front Med (Lausanne). 2024 May 28;11:1382836. doi: 10.3389/fmed.2024.1382836 (PMC11165061; doi:10.3389/fmed.2024.1382836)
Supplement: Supplementary file 1 [file Data_Sheet_1.ZIP › data/GCST90274798_5e6/798.leaveoneout.pdf]

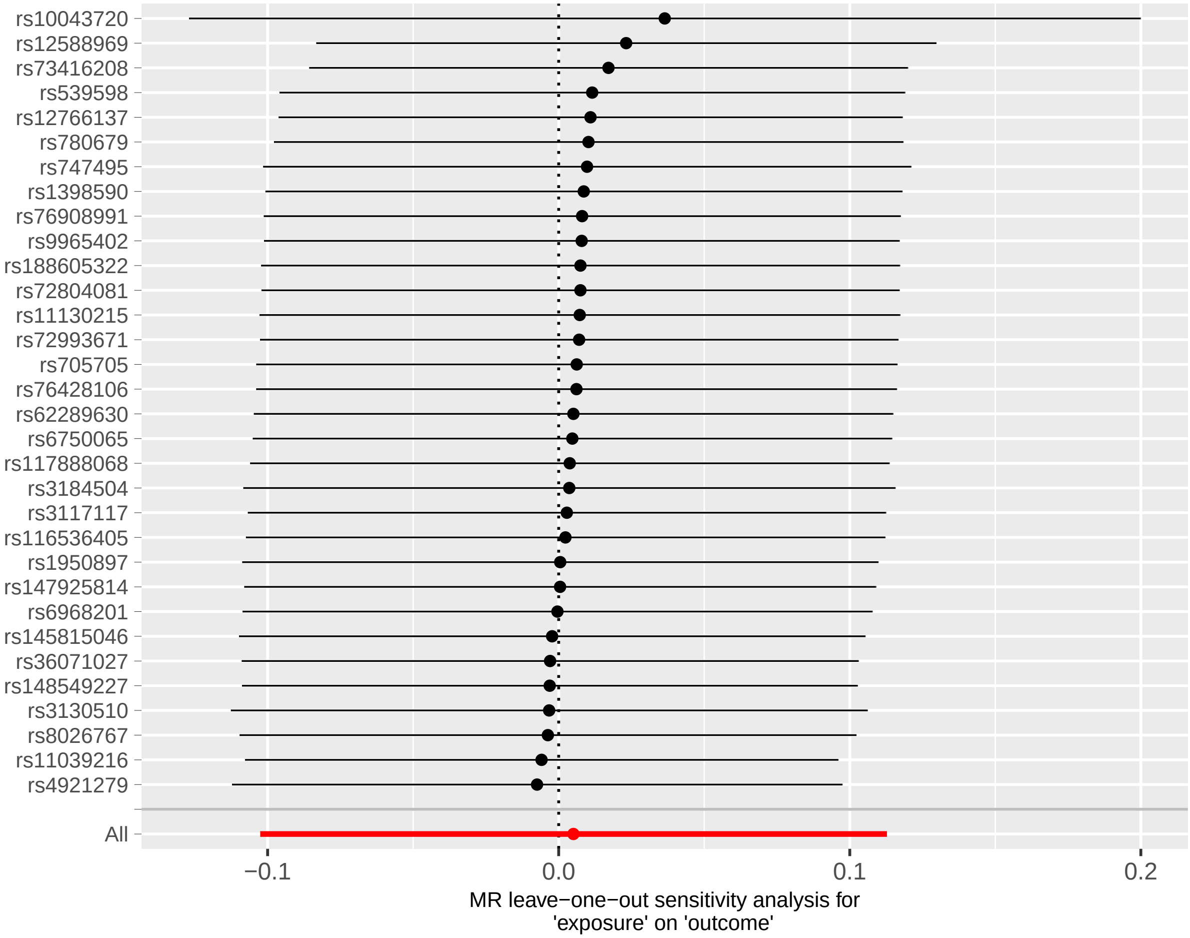
rs10043720
rs12588969
rs73416208
rs539598
rs12766137
rs780679
rs747495
rs1398590
rs76908991
rs9965402
rs188605322
rs72804081
rs11130215
rs72993671
rs705705
rs76428106
rs62289630
rs6750065
rs117888068
rs3184504
rs3117117
rs116536405
rs1950897
rs147925814
rs6968201
rs145815046
rs36071027
rs148549227
rs3130510
rs8026767
rs11039216
rs4921279
All
−0.1
0.0
0.1
0.2
MR leave−one−out sensitivity analysis for
'exposure' on 'outcome'
